# Phylogeography of hepatitis B virus: The role of Portugal in the early dissemination of HBV worldwide

**DOI:** 10.1371/journal.pone.0276618

**Published:** 2022-12-22

**Authors:** Rute Marcelino, Ifeanyi Jude Ezeonwumelu, André Janeiro, Paula Mimoso, Sónia Matos, Veronica Briz, Victor Pimentel, Marta Pingarilho, Rui Tato Marinho, José Maria Marcelino, Nuno Taveira, Ana Abecasis

**Affiliations:** 1 Centro de Investigação Interdisciplinar Egas Moniz (CiiEM), Instituto Universitário Egas Moniz, Caparica, Portugal; 2 Faculdade de Farmácia, Research Institute for Medicines (iMed.ULisboa), Universidade de Lisboa, Lisboa, Portugal; 3 Global Health and Tropical Medicine (GHTM), Instituto de Higiene e Medicina Tropical/Universidade Nova de Lisboa (IHMT/UNL), Lisboa, Portugal; 4 AIDS Research Institute-IrsiCaixa and Health Research Institute Germans Trias i Pujol (IGTP), Hospital Germans Trias i Pujol, Universitat Autònoma de Barcelona, Badalona, Spain; 5 Faculdade de Medicina de Lisboa, GenoMed–Diagnósticos de Medicina Molecular, Instituto de Medicina Molecular, Lisboa, Portugal; 6 Laboratory of Viral Hepatitis, National Center for Microbiology, Institute of Health Carlos III, Madrid, Spain; 7 Department of Gastroenterology and Hepatology, Santa Maria Hospital, Medical School of Universidade de Lisboa, Portugal; Centers for Disease Control and Prevention, UNITED STATES

## Abstract

In Portugal, the genetic diversity, origin of HBV and the Portuguese role in the dissemination of HBV worldwide were never investigated. In this work, we studied the epidemic history and transmission dynamics of HBV genotypes that are endemic in Portugal. HBV pol gene was sequenced from 130 patients followed in Lisbon. HBV genotype A was the most prevalent (n = 54, 41.5%), followed by D (n = 44, 33.8%), and E (n = 32, 24.6%). Spatio-temporal evolutionary dynamics was reconstructed in BEAST using a Bayesian Markov Chain Monte Carlo method, with a GTR nucleotide substitution model, an uncorrelated lognormal relaxed molecular clock model, a Bayesian skyline plot, and a continuous diffusion model. HBV subgenotype D4 was the first to be introduced in Portugal around 1857 (HPD 95% 1699–1931) followed by D3 and A2 a few decades later. HBV genotype E and subgenotype A1 were introduced in Portugal later, almost simultaneously. Our results indicate a very important role of Portugal in the exportation of subgenotypes D4 and A2 to Brazil and Cape Verde, respectively, in the beginning of the XX century. This work clarifies the epidemiological history of HBV in Portugal and provides new insights in the early and global epidemic history of this virus.

## Introduction

Hepatitis B virus (HBV) infection is a severe life-threatening disease with an estimated prevalence, in 2019, of 296 million infections worldwide and a reported death toll of 820,000 individuals due to complications related to HBV chronic infection, such as liver cirrhosis and hepatocellular carcinoma [1]. Despite the existence of an effective vaccine since 1982 and potent antiviral drugs HBV infection remains a serious public health concern [1].

HBV belongs to the Hepadnaviridae family and like all the viruses from this family it causes a hepatotropic infection that drives a lifelong dynamic disease, where the immunological response of the host against the virus induces an ongoing liver inflammation process. This inflammation increases the risk of fibrosis, cirrhosis, end-stage liver disease and hepatocellular carcinoma [2]. The clinical outcomes may vary due to different host and viral factors, such as gender, age, ethnicity, quantitative HBs antigen (HBsAg) levels, viral load, specific mutations of the virus and HBV genotype [3–5].

There are nine viral genotypes (A to I), having a pairwise genomic divergence of at least 7.5%. Genotypes comprise several subgenotypes having a pairwise divergence of 4% to 7.5%. Over the years, the discovery of new subgenotypes has been extensively described [6–10], but there have been many debates about the possible misclassification of some of them [11]. In the absence of an ICTV classification, several attempts have been made to standardize the ICTV classification system [12–15]. Although there are still many debates on the subject, two of the most detailed studies on the classification of subgenotypes report that there are three A subgenotypes A1, A2, A4, and quasi subgenotype A3, six B subgenotypes B1, B2, B4–B6, and quasi subgenotype B3, six C subgenotypes C1–C16, six D subgenotypes D1–D6, four F subgenotypes F1-F4 and two I subgenotypes I1 and I2 [12]. A potential 10^th^ genotype, genotype J, was found to be a recombinant of genotype C and gibbon HBV in the S region and that may represent an independent cross-species transmission [14,16]. The high HBV genetic diversity occurs due to the lack of proofreading activity of the viral reverse transcriptase (RT) enzyme used in its replication process. This makes the virus evolve extremely fast, with an estimated evolutionary rate of about 10^−4^–10^−6^ nucleotide substitutions/site/year (s/s/y). This represents an evolutionary rate around 100 times higher than any other DNA virus [5].

In Europe, vaccination campaigns seem to have contributed to a decline in HBV prevalence [17]. Portugal is a low endemicity country with an HBsAg prevalence in the general population that decreased along the years until achieving 0.02–1.45% in the decades of 1990–2014 [18–20]. Nonetheless, 2.9% of hospital admissions for liver cirrhosis in Portugal, between 2003 and 2012, were related to hepatitis B [21].

Limited information exists regarding HBV diversity in Portugal. Between 2004 and 2014 genotype D was the most prevalent genotype in chronically infected Portuguese patients [20]. Genotype E was referred as more common in Central-Southern (10–62%) than in Northern Portugal (1–4%) [18–22]. Genotypes C and F have been reported in the North of the country on rare occasions [23]. In addition to this, previously unpublished data of a small patient population of Hospital Santa Maria in Lisbon indicated the circulation of minority genotypes such as B, C and F [24]. Historical migration patterns to and from Portugal may have shaped the epidemiological profile of HBV infection in the country and, therefore, it is important to investigate the epidemiological history, transmission dynamics and origin of HBV strains circulating in Portugal. The aim of this study was to characterize the epidemic history of HBV genotypes in Portugal as well as understanding Portugal’s role in the spread of HBV around the world, through the reconstruction of the spatiotemporal evolutionary dynamics of the virus using a Bayesian framework.

## Materials and methods

### Study participants and ethics statement

In this retrospective study we used plasma samples collected for diagnostic purposes from 2005 to 2012 from chronic HBV Portuguese patients assisted in Department of Gastroenterology and Hepatology of Santa Maria Hospital, Medical School of Lisbon, Portugal. Samples (N = 130) were selected for sequencing based on sample volume and the availability of information on age and gender. This study was approved by the Institutional Review Board of Santa Maria Hospital (ref. 600/15 of January 7^th^, 2016) and followed all ethical principles of the Declaration of Helsinki.

### Sample processing

For diagnostic purposes, our laboratory received peripheral blood samples from all patients. The samples were centrifuged at 1500 rotations per minute (rpm) for 15 minutes and the plasma was collected into one or more properly labeled 5ml polypropylene tubes and immediately stored at -80°C. Among all samples received over the years, 130 were selected for this study as described in the above section.

### HBV genotyping

Viral nucleic acid was extracted from 200 μl of plasma using the QIAamp DNA Blood Mini Kit (Qiagen, USA). Following our homemade protocol (available at protocols.io), a 943 base pair (bp) fragment of the pol gene region was amplified by nested PCR with the BIOTAQ DNA polymerase (Bioline, United Kingdom) using the following primer pairs (positions based on the complete reference sequence NC_003977): 5’-ATCCTCACAATACCGCAGA-3’ (position: 229–247) and 5’-AGGAGTTCCGCAGTATGG-3’ (position: 1285–1268) (First round); 5’-AGACTCGTGGTGGACTTCTCT-3’ (position: 252–272) and 5’-GCGTCAGCAAACACTT-3’ (position: 1195–1180) (Second round). The thermocycling conditions for both PCR rounds were: 1 cycle at 94°C for 4 min, and 40 cycles at 94°C for 45 sec; 55°C for 30 sec; 72°C, 1 min with increments of 5 sec/cycle. A final elongation step of 15 min at 72°C was included at the end of amplification run. Amplified DNA products were sequenced on the automated sequencer ABI-3130XL (Applied Biosystems, USA) using the second PCR round primer pair and BigDye terminator v. 3.1 cycle sequencing kit (Applied Biosystems, USA). The sequences were analyzed using Chromas Pro.1.7.6 software (Technelysium, Pty, Australia). Multiple sequence alignment was performed using the ClustalW algorithm, as implemented in the software ClustalX 2.1 [25]. Genomic sequences were genotyped using the Geno2Pheno HBV online tool (https://hbv.geno2pheno.org/). The reliability of the Geno2Pheno results was confirmed by phylogenetic analysis. All the steps described above were carried out in our laboratory.

The HBV Portuguese sequences have been deposited in GenBank under accession numbers:OP796513—OP796642.

### Genetic distances

Evolutionary distances between and within groups were estimated using the Kimura 2-parameter evolutionary model, by calculating the average evolutionary distance between all pairs of groups and between all pairs of sequences within groups, respectively. Standard error estimates were obtained using a bootstrap procedure (1000 replicates). All analyses were conducted in MEGA7 [26]. Rate variation among sites was modelled with a gamma distribution (shape parameter = 1).

### Control sequence selection for spatiotemporal analysis

Control sequences for the spatiotemporal analysis were obtained from GenBank using the NCBI Nucleotide Blast (https://blast.ncbi.nlm.nih.gov/) algorithm. For each of the Portuguese sequences, we retrieved the 50 most similar HBV sequences available in GenBank, making a total of 6500 HBV sequences for the control group. Multiple sequence alignment was performed on this group of 6500 sequences using ClustalW, as implemented in the ClustalX 2.1 software [25]. Duplicate sequences were removed from this dataset with Python v3.7.0 (https://www.python.org/downloads/release/python-370/), reducing the data file to 687 HBV control sequences. From this group, 286 sequences without collection date and country of origin were excluded, creating a data set with 401 control sequences. We tried to keep the temporal and spatial information as wide as possible in the control sequence dataset, diversifying as much as possible both the year of harvest and the country of origin of the control sequences. A manual search was carried out in NCBI, selecting the sequences by genotype and year of harvest and/or country to increase the representativeness of the 60s, 70s and 80s for most genotypes. We also had to increase the representativeness of the origin countries of A1 and D4 subgenotypes, to ensure that Brazil was not falsely over-represented in alignment since the result of the initial BLAST nucleotide resulted in near 35% of sequences from that country. With this approach, the final dataset consisted of 453 HBV control sequences spanning from 1963 to 2018 (S1 Table) and 130 Portuguese HBV sequences. The sequences were aligned as described above and end gaps were removed. The final sequence alignment was 749 bp in length.

### Spatiotemporal evolutionary dynamics

To minimize the effect of convergent evolution, nine codon positions associated with treatment selective pressure were removed from the alignment. Time-scaled phylogeny, evolutionary rate and phylogeography were estimated using a Bayesian Markov Chain Monte Carlo (MCMC) method implemented in the BEAST v1.10.4 [27], with the GTR+F+R4 nucleotide substitution model that was selected using Model Finder module [28] within IQ-TREE 1.6.11 software [29] and the Akaike information criterion [30].

Eight different evolutionary models were tested: strict vs relaxed molecular clock, each one combined with either parametric (expansion Growth and exponential Growth) and non-parametric (Bayesian Skygrid and Bayesian Skyline) demographic priors and with a continuous Cauchy RRW phylogeography model [31] to reconstruct viral spatio-temporal diffusion. For this continuous model, the midpoint of latitude and longitude obtained from google maps were used as coordinates for each sequence origin country. In each case, two independent MCMC chains were run for 300 million generations (sampled every 30.000 steps) to ensure convergence and Effective Sample Size (ESS) values > 100. Our data only converged and showed acceptable ESS values when using an uncorrelated lognormal relaxed molecular clock model under a Bayesian skyline plot (a non-parametric piecewise-constant model) as coalescent priors.

Uncertainty of parameter estimates was assessed after excluding the initial 10% of the run by calculating the 95% Highest Probability Density (HPD) values using TRACER v1.7.1 program [32]. Maximum clade credibility (MCC) tree was summarized from the posterior distribution of trees with TreeAnnotator included in BEAST package and visualized and annotated with FigTree v1.4.4 (http://tree.bio.ed.ac.uk/software/figtree/) to show node support values (posterior state probability) and median time of MRCAs with High Posterior Density 95% for each common ancestral node. Bifurcating nodes with posterior probability greater than 0.90 were considered statistically well supported.

## Results

### Genotype A was the most prevalent in Portugal

Most (n = 97, 74.6%) of the patients included in the study were male with a median age of 44 years. Median age of females (n = 33, 25.4%) was 40 years (S2 Table). According to Geno2Pheno genotype classification, HBV genotype A (HBV/A) was the most prevalent genotype (n = 54, 41.5%), followed by D [HBV/D; (n = 44, 33.8%)], and E [HBV/E; (n = 32, 24.6%)]. Subgenotypes A1 (HBV/A1) and D4 (HBV/D4) were almost equally prevalent with 23.1% (n = 30) and 22.3% (n = 29), respectively, followed by A2 (HBV/A2) with 16.2% (n = 21) and D3 (HBV/D3) with 11.5% (n = 15). Three sequences (n = 2.3%) were classified as genotype A without further subgenotype classification.

Genetic distances between HBV genotypes and subgenotypes present in Portugal were >7.5% for genotypes and between 4%-7.5% for subgenotypes, consistent with rules of classification (Table 1).

**Table 1:** Divergence between HBV genotypes/subgenotypes present in Portugal.

	Sub GT_A1	Sub GT_A2	Sub GT_D3	Sub GT_D4	GT_E
Sub GT_A1		0.041	0.091	0.086	0.086
Sub GT_A2	**0.006**		0.097	0.088	0.096
Sub GT_D3	**0.011**	**0.011**		0.049	0.082
Sub GT_D4	**0.011**	**0.011**	**0.007**		0.074
GT_E	**0.010**	**0.011**	**0.010**	**0.010**	

The number of base substitutions per site from averaging over all sequence pairs between groups are shown above the diagonal and standard error estimate(s) are shown below the diagonal in bold.

Genetic divergence within genotypes/subgenotypes varied between 1.7% for genotype E and 2.3% for genotype A1 which is consistent with the higher prevalence of this genotype in Portugal (see below in Table 2).

**Table 2:** Divergence within HBV genotypes/subgenotypes isolates present in Portugal.

Genotype/Subgenotype	Genetic distance	Standard Error
**A1**	0.023	0.003
**A2**	0.018	0.002
**D3**	0.022	0.003
**D4**	0.018	0.002
**E**	0.017	0.002

The number of base substitutions per site from averaging over all sequence pairs within groups are shown in the central column and standard error estimate(s) are shown in the right column.

### Date and origin of HBV genotypes in Portugal

The estimated mean substitution rate for HBV was 9.45x10^-5^ s/s/y (95% HPD: 4.22x10^-5^–1.43x10^-4^). Data from spatiotemporal reconstruction analysis suggest that HBV may have originated in France (posterior state probability (PSP) = 1) around the year 886 (95% HPD: 414 B.C—1514) (Table 3).

**Table 3:** Estimated time and place of HBV origin, origin of the Portuguese strains, time of introduction in Portugal and role of Portugal in the global HBV spread.

Event	Median year [HPD 95%]	PSP	Country of origin	% of PT clustered sequences (N)		Spread from Portugal to
HBV origin	886 [414 B.C—1514]	1	France			
**GT A emergence**	1537 [1037–1787]	1	France			
GT A arrival in Portugal	1933 [1864–1967]	0.9	Mali	3.03% (1)		N.O.
	1950 [1887–1981]	0.36	Cameroon	3.03% (1)		N.O.
	1932 [1851–1975]	0.98	Spain	3.03% (1)		Cuba
A1 emergence	1711 [1416–1855]	0.98	Libya			
A1 arrival in Portugal	1913 [1834–1952]	0.17	Brazil	78.8%	42.3% (11)	France, Italy, Netherlands and Cape Verde
	1909 [1820–1954]	0.29	Brazil		57.7% (15)	France, Italy and Germany
	1954 [1894–2002]	0.95	Zimbabwe	6.06% (2)		N.O.
	1923 [1846–1963]	0.72	Algeria	6.06% (2)		India
A2 emergence	1785 [1559–1891]	1	Belgium			
A2 arrival in Portugal	1883 [1767–1940]	1	Belgium	85.7%	38.9% (7)	Spain, Germany and France and Cape Verde
	1925 [1913–1936]	0.0	Belgium		61.1% (11)	Cuba and Cameron
	1944 [1885–1974]	0.91	Spain	14.3% (3)		N.O
**GT D emergence**	1501 [922–1790]	0.99	France			
D3 emergence	1779 [1547–1897]	1	France			
D3 arrival in Portugal	1882 [1756–1933]	0.01	France	40% (6)		Brazil, USA and Spain
	1899 [1811–1944]	0.5	France	60% (9)		Spain, Brazil and back to France
D4 emergence	1766 [1523–1885]	1	France			
D4 arrival in Portugal	1857 [1699–1931]	0.47	France	100%		Brazil, Germany and back to France
**GT E emergence**	1818 [1619–1914]	1	France			
GT E arrival in Portugal	1912 [1835–1948]	0.16	Spain	50% (16)		USA
	1906 [1811–1951]	0.46	Spain	50% (16)		Cape Verde and Norway

N.O, no occurrence; PSP, posterior state probability; GT, genotype; PT seq, Portuguese sequences; B.C., before Christ (calendar years not labelled with B.C. should be considered After Christ.

**Fig 1:**
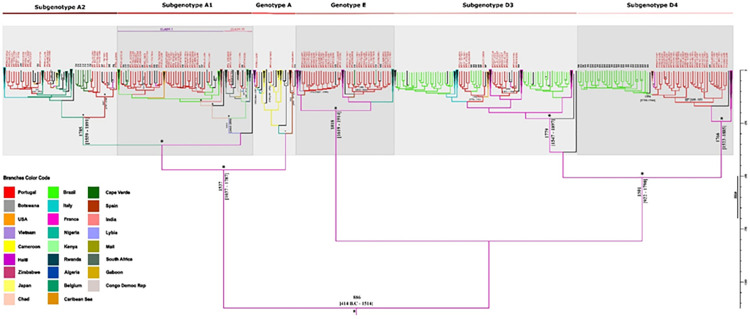
Bayesian maximum clade credibility tree of HBV partial polymerase gene sequences. The colored internal branches represent the locations of the parental nodes (based on node latitude and longitude coordinates), as assigned by continuous Bayesian phylogeography (color code below the tree). Each branch color allows to perceive the dispersion patterns of each genotype from a node (location) to another. Branches and tips of Portuguese isolates are labeled in red. External branches labeled with black initials represent the countries to where Portugal exported HBV strains (BR-Brazil; CM-Cameroon; CU-Cuba; CV-Cape Verde; DE-Germany; FR-France; IN-India; IT-Italy; NL-Netherlands; NO-Norway; SP-Spain; US-United States of America). The external branches representing Portuguese speaking countries, Brazil and Cape Verde, are highlighted in light green and dark green, respectively. Collapsed branches (triangles) represent clades that are not of primary interest for the study and are too large for displaying in full. They are filled in black because they include isolates from different origins, being outlined with the color of the country that originated them. Within subgenotype A1, Clade I–A1 Asian/American clade is labeled with a purple line, while Clade II–A1 African Clade is labeled with a rose line. The scale on the right of the tree represents years before the last sampling time (2012). Tree is annotated with genotypes/subgenotypes emergence dates and dates of introduction in Portugal. Branches with a posterior state probability support ≥0.9 are annotated with *. More information about control reference sequences (origin, date of collection and accession number) is available in S1 Fig as well as in S1 Table in support information.

It diverged into one branch that gave rise to genotype A and another that originated the remaining genotypes (Fig 1).

Among the studied genotypes, HBV/D was probably the first genotype to emerge in France around 1501, branching off into subgenotypes D3 and D4 likely in the second half of XVIII century (Fig 1; Table 3).

In Portugal, HBV/D4 was the first to emerge possibly due to a single introduction estimated to have occurred around 1857 via France. Similarly, HBV/D3 was introduced in Portugal via France first in 1882 and later in 1899. HBV/A2 has possibly emerged in Belgium near 1785 and was introduced in Portugal in 1883 and in 1925, via Belgium, and in 1944, via Spain. (Fig 1; Table 3). HBV/A1 likely has its origin in the region of Libya in North Africa around 1711 (Fig 1; Table 3) and migrated across countries such as Chad. From this region, it seems to have been introduced in Brazil and India near 1869, giving rise to a group of sequences related to clade I, known as the Asian-American clade (Fig 1). Finally, coming from Brazil, it was introduced in Portugal around 1909 and 1913 (Fig 1; Table 3). A second route of A1 subgenotype introduction in Portugal related to clade II, known as African clade, occurred directly via Africa, possibly through Algeria around 1923 and Zimbabwe around 1954 (Fig 1; Table 3). Our data indicates that genotype E probably emerged more recently than all the others near 1818 in France (Fig 1; Table 3). After migrating to Spain, it was introduced in Portugal around 1906 and 1912 creating two subclusters of Portuguese sequences (Fig 1). Finally, the three strains to which no subgenotype A classification was assigned had different origins. While one entered Portugal around 1932 via Spain, the other two strains had an African origin arriving to Portugal around 1933 and 1950 (Fig 1, Table 3).

### Global exportation of HBV from Portugal

**Fig 2:**
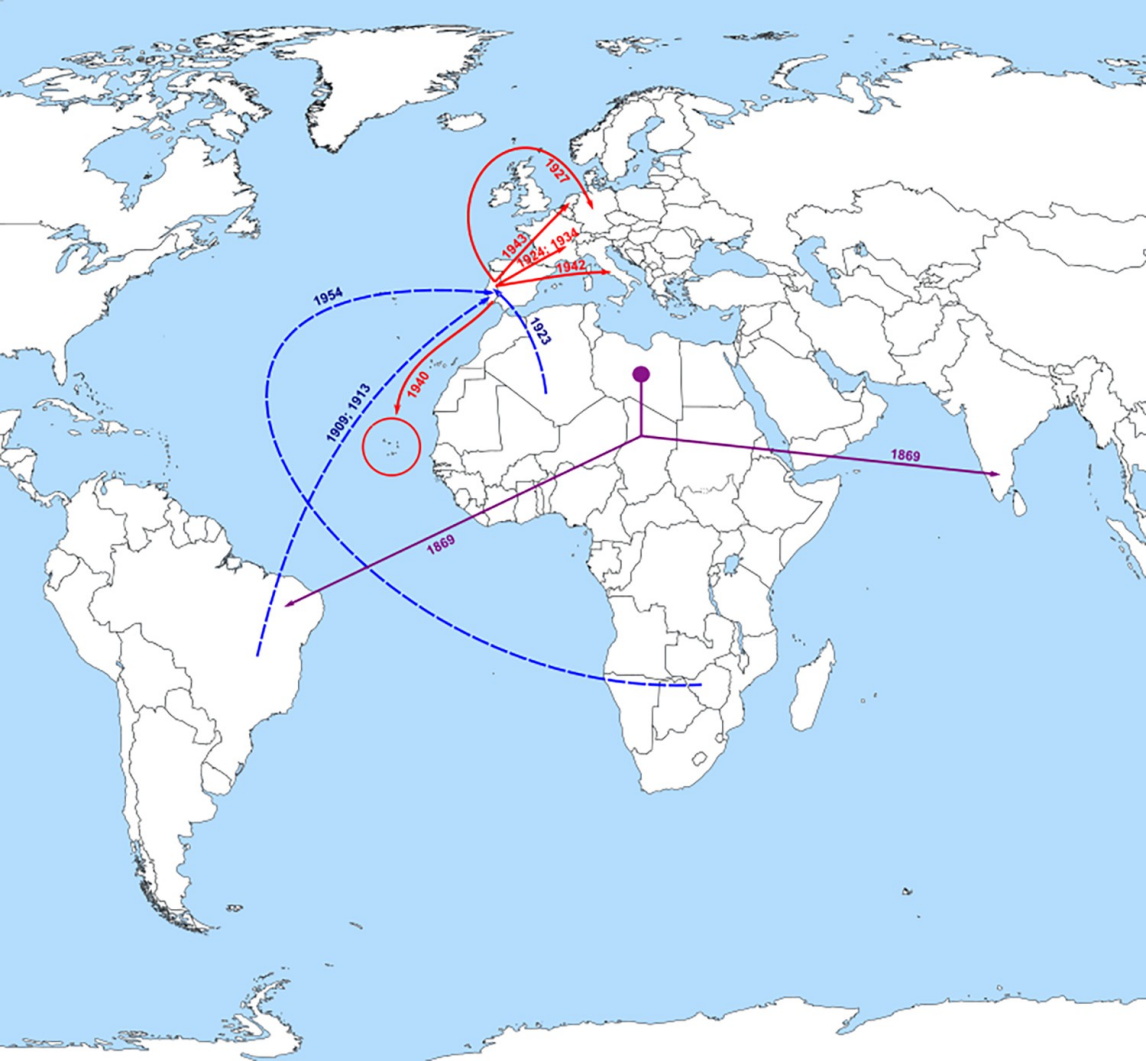
Probable major dispersal pathways of HBV/A1 as estimated by phylogeographic analysis. The dashed blue arrows represent probable routes of introduction of the HBV/A1 in Portugal. The solid red arrows represent the probable dispersion paths of HBV/A1 from Portugal towards other countries. A significant dispersion event, prior to the introduction of A1 in Portugal, is represented in purple: The purple circle is located on the probable country of emergence of HBV/A1 indicating the out of Africa dissemination direction that seemingly occurred simultaneously to Brazil and India. This figure is a derivative of “World map blank with blue sea.svg” licensed under the Creative Commons Attribution-Share Alike 4.0 International, modified with the addition of dates, dashed blue and solid red arrows.

HBV/A1 spread from Portugal to other countries in Europe, mainly Italy (1942) and France (1924 and 1934), as well to Cape Verde Islands in Africa (1940). Less frequently HBV/A1 spread to Germany (1927) and Netherlands (1943) (Figs 1 and 2).

**Fig 3:**
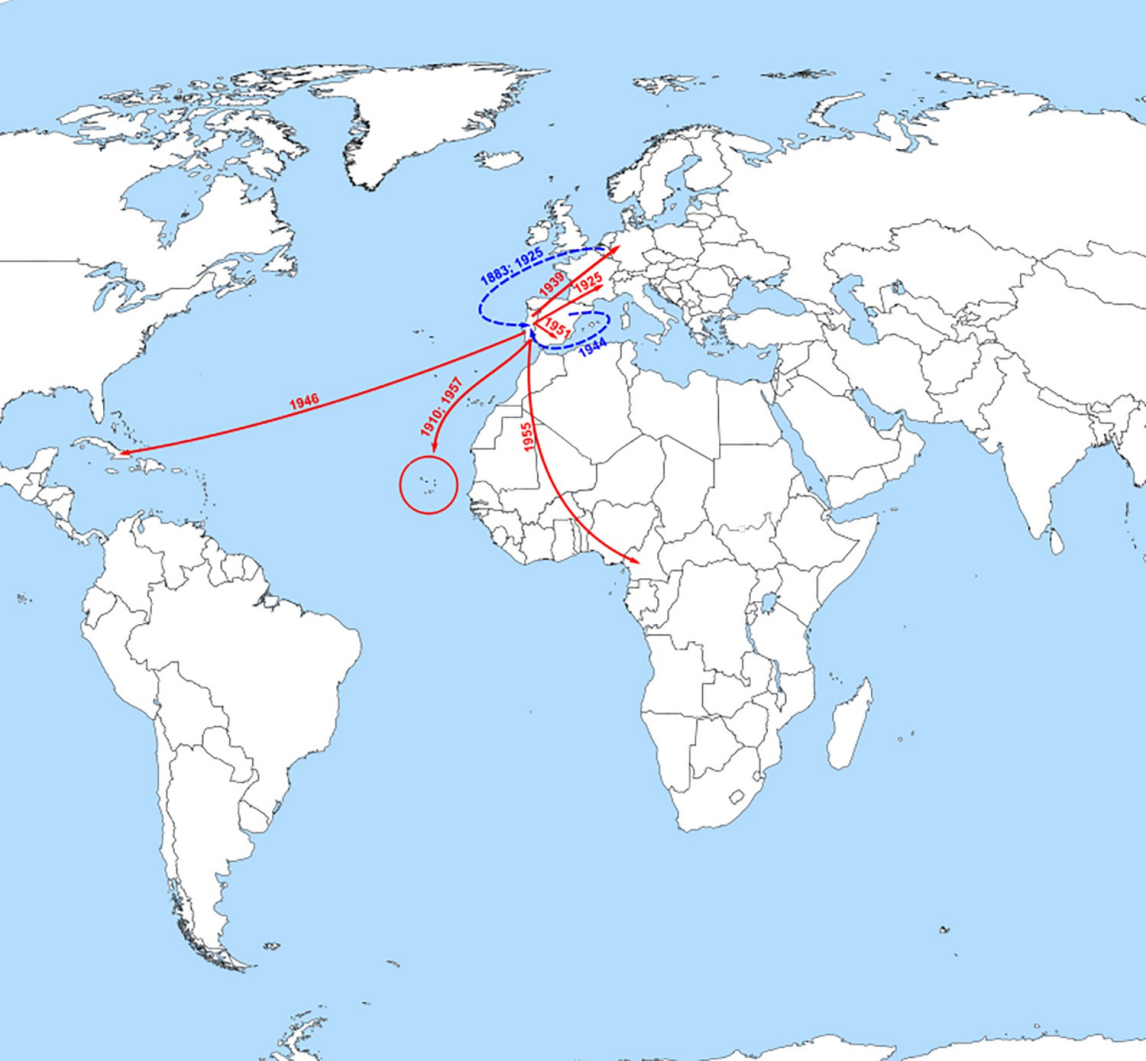
Probable major dispersal pathways of HBV/A2 as estimated by phylogeographic analysis. The dashed blue arrows represent probable routes of introduction of the HBV/A2 in Portugal. The solid red arrows represent the probable dispersion paths of HBV/A2 from Portugal towards other countries. This figure is a derivative of “World map blank with blue sea.svg” licensed under the Creative Commons Attribution-Share Alike 4.0 International, modified with the addition of dates, dashed blue and solid red arrows.

HBV/A2 spread to Spain, Germany and France (1951, 1939 and 1925, respectively) and to Cuba (1946) and African countries such as Cameroon (1955) and Cape Verde (1910 and 1957) (Figs 1 and 3).

**Fig 4:**
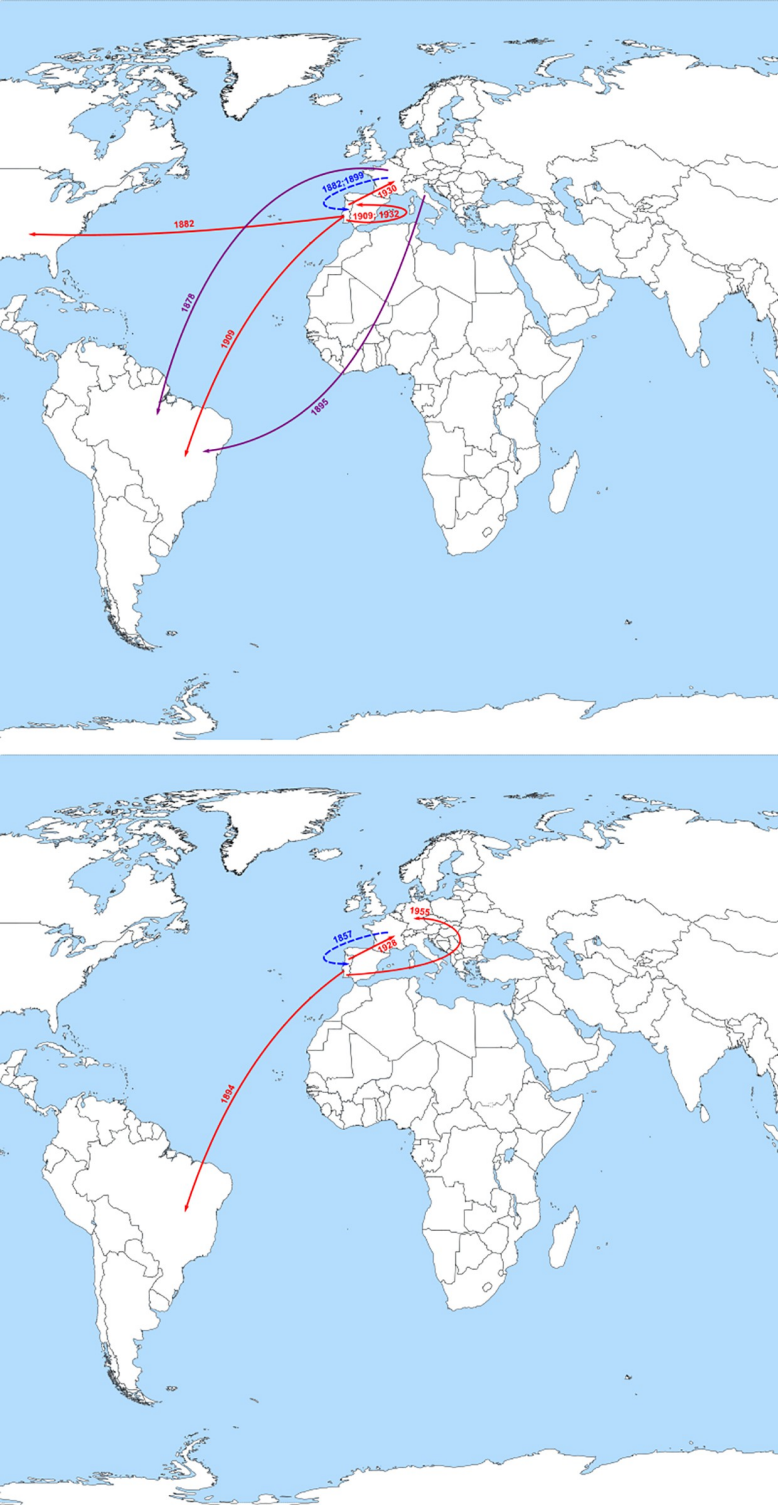
Probable major dispersal pathways of subgenotype HBV/D3 (above) and HBV/D4 (below) as estimated by phylogeographic analysis. The dashed blue arrows represent probable routes of introduction of the subgenotypes in Portugal. The solid red arrows represent the probable dispersion paths of subgenotype from Portugal towards other countries. Purple arrows show the contribution of other countries, besides Portugal, to the introduction of D3 subgenotype in Brazil. This figure is a derivative of “World map blank with blue sea.svg” licensed under the Creative Commons Attribution-Share Alike 4.0 International, modified with the addition of dates, dashed blue and solid red arrows.

As mentioned above, HBV/D3 entered Portugal via France around the year of 1882 and then was exported to Brazil near 1909, while also spreading to USA (1882) and Spain (1932) (Figs 1 and 4).

The HBV/D3 strains introduced in Portugal near 1899 were also exported both to Brazil and Spain near 1909. From Spain they were later introduced again in Portugal from where they spread to Germany and Cuba, likely via France (Fig 1). Other countries exporting HBV D3 to Brazil were France (1878) and Italy (1895) (Figs 1 and 4).

Subgenotype D4 was exported to Brazil around 1894, generating a cluster of Brazilian HBV/D4 strains. Portugal also exported HBV/D4 to France (1928) and Germany (1955) (Figs 1 and 4).

**Fig 5:**
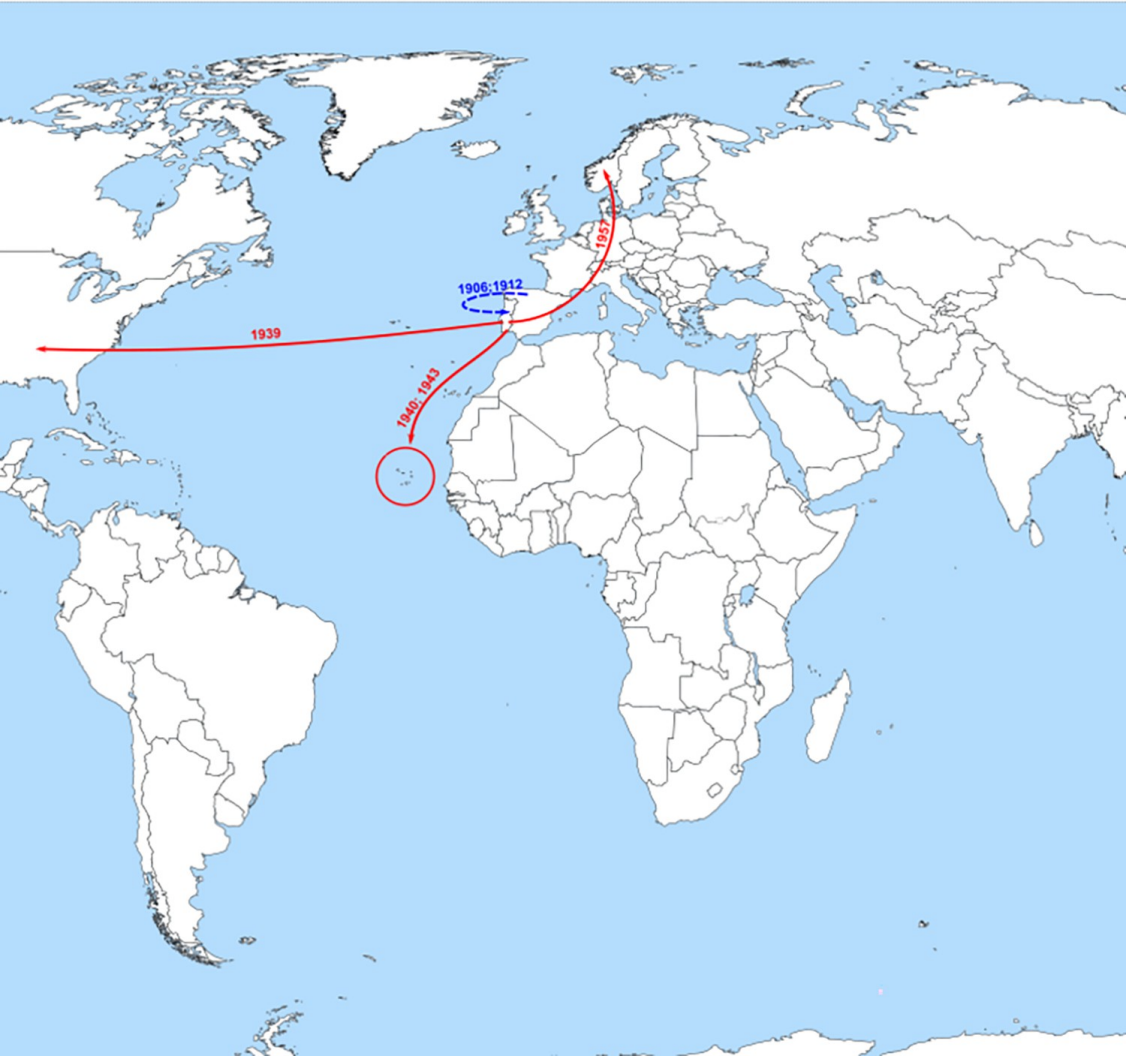
Probable major dispersal pathways of HBV/E as estimated by phylogeographic analysis. The dashed blue arrows represent probable routes of introduction HBV/E in Portugal. The solid red arrows represent the probable dispersion paths of HBV/E from Portugal towards other countries. This figure is a derivative of “World map blank with blue sea.svg” licensed under the Creative Commons Attribution-Share Alike 4.0 International, modified with the addition of dates, dashed blue and solid red arrows.

Finally, one of the Portuguese HBV/E clusters seems to have been exported twice to Cape Verde (1940 and 1943) and Norway (1957), while the other has been spread to the USA (1939) (Figs 1 and 5).

## Discussion

This study examined the genotypes of HBV circulating in a major hospital of Lisbon, Portugal, between 2005 until 2012 using phylogenetic analysis, and determined the spatial and temporal origin of HBV genotypes in Portugal as well as their global diffusion out of Portugal using a Bayesian framework. Genotype A strains prevailed over D and E which was the least common genotype. Previous studies done in 2008–2009 and 2013–14 in the north or center of the country, with samples from prisoners, pregnant women, or blood donors reported genotype D as the most prevalent [18–23]. However, none of these studies used phylogenetic analysis to classify the HBV strains. While, the higher prevalence of genotype A strains in the current study may be associated in part with the use of a different method to classify HBV strains, the results may also reflect a sampling effect since during the period of sample collection for this study since there was a high influx of migrants from Cape Verde and Brazil to Lisbon [33] and HBV/A genotypes prevail in these countries [34,35].

Molecular-clock-based analysis has been used to reconstruct the evolution of HBV as well as dating its origin, but the results have proven to be unclear and controversial [36–38]. Some studies have dated the origin of HBV as recent as 400 years ago [36,38,39] and other as old as more than 7000 years ago [40]. In this study an estimated mean substitution rate of 9.45x10^-5^ s/s/y (95% HPD: 4.22x10^-5^–1.43x10^-4^) was obtained, consistent with other recent studies [35,41–44]. One of the main limitations is that we did not analyze each HBV genotype independently. However, while this could pertain us from obtaining reliable estimates of the Bayesian evolutionary parameters, such estimations are plausible given the flexibility of all the evolutionary, demographic, molecular clock and phylogeographic models that were used in the analysis. Furthermore, we confirmed that the ESS values sufficiently converged in replicate runs when these multiple HBV genotypes were combined, allowing estimation of evolutionary rates and tMRCAs comparable to what has been obtained in previous studies.

Our data traced back the origin of HBV to the present region of France around the year 886 (Medieval Age) with a highest posterior density interval between the years 414 B.C (Ancient Age) and 1514 (Modern Age). There is recent evidence of the presence of HBV in Medieval and Neolithic human remains from Europe, proving that HBV is an ancient virus in this continent [39]. However, France as origin location of the HBV still seems less plausible. This disparity of our results could be due to the fact that we analyzed all genotypes together or that no ancient sequences were introduced during the construction of our control dataset. Kocher et al., using HBV infected human remains that dated back to 10,000 years ago estimated the tMRCA of all HBV lineages to between ~20,000 and 12,000 years ago [45]. In contrast, the oldest sample in our study originated from 1963, making it plausibility that there is an underestimation of the tMRCA of HBV in our study. On the other hand, we don’t expect that the estimated dates of the tMRCAs of HBV genotypes are largely biased, as the relaxed clock is much more accurate to date more recent splitting events.

Due to this limitation of our study, our focus is not the origin of HBV but only to describe the epidemic history of the specific HBV GT and sub-GT circulating in Portugal (HBV/A, HBV/D and HBV/E).

In agreement with Kramvis and Paraskevis [40], in our study the origin of HBV/A1 was traced back to Africa. However, contrarily to Kramvis’ suggestion that pointed to Southern Africa as origin of subgenotype A1, or even another theory that proposed East Africa as the origin [46], our data raises the hypothesis that the origin was in North Africa. HBV/A1 evolution seems to have occurred not just along the North of the continent and Chad, from where it appears to have been exported directly to Brazil and India (clade I), but also in Kenya. From this region, it dispersed across Eastern, Central and Southern Africa (clade II), and then to Europe via Portugal and Belgium (see S1 Fig). These two distinct clades correspond to the previously described Asian-American and African clades, respectively [40].

A previous study hypothesized that an alternative route to the slave trade could explain the fact that Brazilian HBV sequences clustered in the Asian-American clade instead of in the African clade, possibly being imported from East Africa or Asia by merchants in the middle of the XIX century [35]. Our results suggest instead, that HBV/A1 dispersed directly from Africa to Brazil, and India, in a period when Portugal was strongly present in both territories, probably contributing with African slaves as working labor to both continents. These facts raise the hypothesis that Portuguese slave trade was at the origin of the Asian-American clade. In addition, our temporal tree revealed that Brazil seemingly exported its strains to other American countries, namely Uruguay, Panama, Haiti, Cuba, USA, and Argentina, while India exported its strains to Japan, China, Bangladesh, and Philippines (see S1 Fig). These historic connections provide a plausible explanation for the evolutionary proximity between HBV isolates from Americas and Asia.

Portugal seems to have imported most of the HBV/A1 strains from Brazil and then exported it to France, Italy, Netherlands, Germany and to Cape Verde, at this time still a Portuguese colony. In fact, the Portuguese role in exporting HBV/A1 into Cape Verde may explain why Cape Verdean HBV/A1 strains are more associated with the Asian-American clade than with the African clade as should be expected [34].

HBV/A2 possibly emerged between the middle of XVI and the end of XIX centuries in Belgium, which seems to have played a main role in the spreading of HBV/A2 in Europe, exporting this subgenotype to Portugal more than once. A relevant role was also played by Portugal in spreading HBV/A2 outside of the European continent, mainly to Cape Verde between the beginning of the XIX and the middle of XX centuries. A2 strains from Cape Verde were previously described to group into a single cluster quite separate from the HBV/A2 strains of the other geographic regions (continental Africa, Asia, the Americas, or Europe), except for a strain from Poland [34], a European country to which Portugal also seemingly exported HBV/A2 via France. Our results provide the first indication that Portugal was the source of HBV/A2 isolates found in Cape Verde.

Evidence from our Bayesian analysis suggests that HBV/D emerged earlier than HBV/A, around the year of 1501, with a confidence interval that covers the vast period from 922 to 1790. Our study points to a putative origin of HBV/D in France, while previous phylogeographic analysis indicated North Africa/Middle East [47], Southern Europe, Central/Eastern Europe, Syria, or Martinique [43] as the putative origin of HBV/D. The lack of HBV/D sequences from more diversified locations in the world and covering similar genomic regions is probably limiting consensus conclusions in phylogeographic studies.

Our results indicate that Portugal played a fundamental role in the dissemination of HBV/D3 and HBV/D4 during the period that covers a great wave of Portuguese emigration to the American continent in the late XIX and early XX centuries.

All Portuguese HBV/D4 sequences clustered together suggesting a single introduction of this subgenotype in our country via France. This Portuguese cluster seems to be the most likely and almost exclusive origin of HBV/D4 infections in Brazil, whose sequences also cluster monophyletically with just one exception. This clustering effect of D4 Brazilian sequences was previously described, and a single introduction of D4 in Brazil had been proposed by the authors with a probable time of introduction of 1848 (CI: 1062–1946) [43]. However, spatial origin was pointed at Martinique, which the authors considered incompatible with epidemiological and historical data of HBV in the Americas [43]. Our results help to further clarify these previous results, now showing that Portugal is at the origin of Brazilian D4 strains with its most recent common ancestor (tMRCA) dating around 1894 (CI:1788 to 1944) which coincides with the period of massive emigration from Portugal to Brazil by the end of XIX century [48]. As the confidence interval still covers the end of the XVIII century, it is not possible to exclude the slave trade factor contributing for the introduction of this subgenotype in Brazil. However, the parallel exportation of HBV/D4 from Portugal to France (1928) and Germany (1955), which were secondary emigration destination of Portuguese people in the first half of last century, further support a later exportation to Brazil and therefore the theory of Portuguese emigration.

Subgenotype D3, introduced in Portugal perhaps a few years later than HBV/D4 (late of XIX century) but also with a probable origin in France, seems to have been exported with Portuguese population together with the Italians and French to Brazil in the beginning of the XX century, around 1909 (95% HPD: 1814–1952). HBV/D3 was previously described to have been introduced in Brazil around 1799 (95% HPD: 1615–1976) by Southern European people [43], especially during the mass European emigration from different countries encouraged by the Brazilian government campaigns, such as Italians, Portuguese, Spanish, Japanese, Germans, Lebanese, and Syrians [49]. The Italians, who were divided between emigration to Europe and Argentina, only came to Brazil in large groups in the last two decades of the XIX century [48], probably introducing genotype D3 in the country as previously proposed [35]. Interestingly, this agrees with our results that indicate that the tMRCA is near 1895 for a group of HBV/D3 Brazilian sequences with origin in an Italian cluster. From the moment the Portuguese people first arrived in South American lands there was always a flow of Portuguese people to the region that became Brazil. Nonetheless, the largest flow of Portuguese emigration started in late XIX and the beginning of XX centuries [48], which agrees with our estimated tMRCA of 1909 for the introduction of Portuguese HBV/D3 in Brazil. The confidence interval of this tMRCA has a lower limit of 1814, which is already very close to Brazil’s independence (in 1822) and consequently to the end of the slave trade by the Portuguese people. This gives more support to the possibility of HBV/D3 introduction in Brazil by the Portuguese to have occurred during the emigration flow in XIX—XX centuries rather than with the slave trade.

Although with less intensity, Portugal also seems to have contributed to the introduction of HBV/D3 subgenotype in the USA, Spain, and France, which agrees with other secondary migratory destinations of Portuguese people [48].

Genotype E has been assumed to have a more recent origin probably in the present region of Nigeria around the last 130 to 200 years, which is supported by its low genetic variability characteristic of a shorter evolutionary history [50,51]. The tMRCA found in our study, 1818, is in full agreement with this. However, contrarily to the theories of these authors, our data indicates France as the origin location for HBV/E instead of Africa. One possibility for this disparity is a bias caused by the fact that some HBV/E sequences available in the databases do not indicate sample collection date and geographical origin and therefore could not be used in our study. Ingasia et al found sequences from West Africa had a location in their Maximum Likelihood tree suggestive that HBV/E epidemic probably originated in the West Africa expanding later to other regions, within and outside Africa. Other evidence of their work pointed that the European HBV/E strains originated mostly from West Africa [52]. The HBV/E control references from our study retrieved from the NCBI do not include many strains from either Nigeria or West African countries due to the limitations above described. For this reason, it is possible that this caused a bias in the location of the origin of HBV/E in our study.

Despite the low genetic variability, HBV/E is hyperendemic throughout West Africa. An explanation for this phenomenon would be iatrogenic transmission in mass-injection campaigns with unsafe injections by the colonial governments [53]. Interestingly, Portugal seems to have imported HBV/E from Spain in the beginning of XX century between the 1880s and 1930s, previously described as the most significant expansion period of this genotype [50]. In this case since Portuguese sequences are not associated with outstanding exportations to other countries. This agrees with the absence of HBV/E in descendants of African slaves in South America [50,54,55]. The absence of the HBV/E from the South American continent also indicates that Europeans who emigrated to the Americas did not introduce it there. This fact reinforces the idea that during the period of emigration from Europe to the American continent, in the end of the XIX century and the beginning of the XX century, HBV/E was probably still absent or weakly incident in Europe.

The main limitation of this study was the inevitable exclusion of the analysis of sequences that were present in databases lacking all the necessary conditions for inclusion in the spatiotemporal reconstruction. Many sequences corresponding to the region of the pol gene under analysis did not have a date of sampling or country of origin and had to be excluded from the analysis. For this reason, some regions of the globe were underrepresented, limiting the analysis. For instance, countries like Angola, Guinea Bissau, Mozambique that share close historical ties with Portugal were not represented equally in the datasets of all the studied genotypes. This might have caused bias in the results. However, regarding HBV/E our analysis is the first to use Bayesian methods to study the dispersion of this genotype with so many non-African genotype E sequences, as such our results on the geographical origin of genotype E should be considered for future studies. On the other hand, herein is that we did not analyze each HBV genotype independently. While this could pertain us from obtaining reliable estimates of the Bayesian evolutionary parameters, the flexibility of all the evolutionary, demographic, molecular clock and phylogeographic models that were used allows us to have confidence in the results. Furthermore, we confirmed that the ESS values sufficiently converged in replicate runs when these multiple HBV genotypes were combined, allowing estimation of evolutionary rates and tMRCAs comparable to what has been obtained in previous studies.

## Conclusions

HBV/D4 was the first subgenotype to be introduced in Portugal around 1857 (HPD 95% 1699–1931) followed by subgenotypes D3 and A2 a few decades later, and genotypes E and A1 more recently. Portugal had a major role in the dispersion of HBV to Africa, America, and Asia. This work provides new insights in the early and global epidemic history of HBV.

## Supporting information

S1 Fig Bayesian maximum clade credibility tree of HBV partial polymerase gene sequences. Complete MCC tree without collapsed branches.
(PDF)

S1 Table HBV Control Sequence details used for the spatio-temporal analysis: Origin country, collection date and accession number. The number of sequences by genotype, country and date of collection can be checked by filtering data in each column header.
(XLSX)

S2 Table Age and gender of patients and their respective HBV SEQ_ID. (PDF)
