# Supplementary figures and images for: Phylogeography of hepatitis B virus: The role of Portugal in the early dissemination of HBV worldwide
Source: PLoS One. 2022 Dec 22;17(12):e0276618. doi: 10.1371/journal.pone.0276618 (PMC9778982; doi:10.1371/journal.pone.0276618)

**Figure S1:** Bayesian maximum clade credibility tree of HBV partial polymerase gene sequences

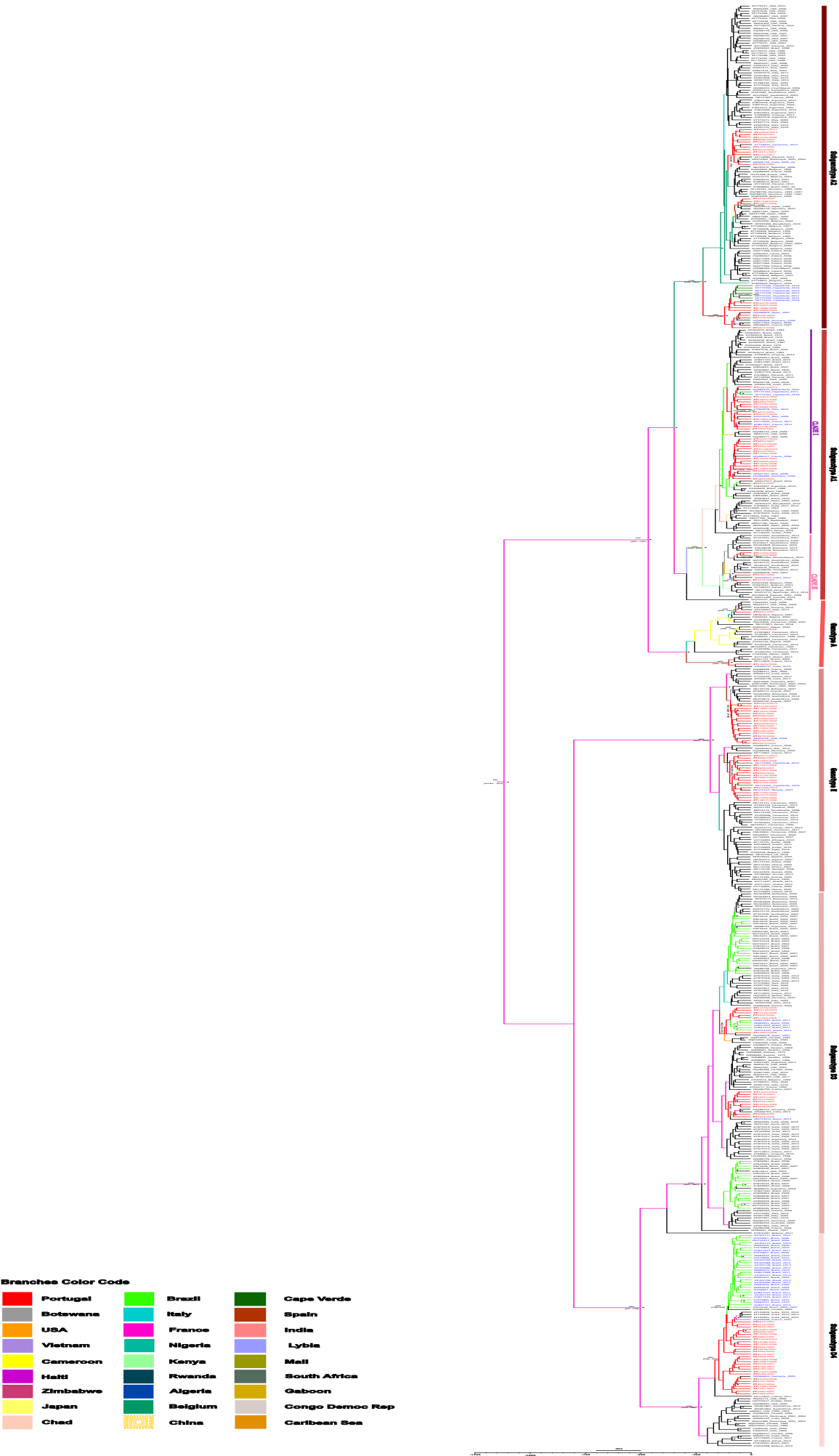

Supplement: S1 Fig — Complete MCC tree without collapsed branches. (PDF) [file pone.0276618.s001.pdf]
