# Supplementary material for: Phylogeography of hepatitis B virus: The role of Portugal in the early dissemination of HBV worldwide
Source: PLoS One. 2022 Dec 22;17(12):e0276618. doi: 10.1371/journal.pone.0276618 (PMC9778982; doi:10.1371/journal.pone.0276618)
Supplement: S2 Table — (PDF) [file pone.0276618.s003.pdf]

S2 Table. Age and gender of patients and their respective HBV SEQ_ID.

SEQ ID	Age	Gender
1942_D4_2005	41	M
2912_E_2005	49	M
2914_A2_2005	38	M
2917_D3_2005	37	M
2947_E_2005	19	M
2948_E_2005	21	M
2980_D4_2005	36	M
3365_D4_2005	35	M
3420_A1_2005	57	F
3421_A1_2005	38	M
3803_A1_2006	64	M
3873_D3_2006	35	M
4017_D4_2006	44	M
4119_E_2006	43	M
4120_A2_2006	63	M
4435_A2_2006	33	M
4771_E_2006	22	M
4774_A1_2006	34	F
4775_A2_2006	35	F
4804_D4_2006	66	M
4934_D3_2006	56	M
4975_E_2006	38	M
4978_A2_2006	31	F
5004_D4_2006	56	F
5088_D3_2006	58	M
5241_A2_2006	29	F
5442_D3_2006	54	M
5611_A2_2006	32	M
5668_D3_2006	36	M
5761_D4_2006	60	F
5930_A1_2006	46	M
6053_E_2006	43	F
6114_D4_2006	50	M
6223_A2_2006	67	M
6781_A1_2006	58	M
7255_E_2007	32	F
7397_D4_2007	45	M
8330_D4_2007	30	F
8605_E_2007	24	M

SEQ ID	Age	Gender
8669_E_2007	51	M
8719_A1_2007	49	M
8760_D4_2007	62	F
8785_D4_2007	35	M
8789_D4_2007	64	M
8877_D4_2007	42	F
8893_A1_2007	36	M
9008_A2_2007	53	M
9042_D4_2007	66	M
9054_D3_2007	42	M
9113_D4_2007	56	M
9172_A2_2007	66	M
9199_E_2007	59	F
9319_D4_2007	61	F
9443_A1_2007	78	M
9456_A1_2007	58	M
9471_A1_2007	31	M
9631_A1_2007	23	F
9632_D4_2007	35	M
9916_A2_2007	53	M
10018_D4_2007	36	F
10067_E_2007	52	M
10188_D4_2007	58	F
10211_A2_2007	42	M
10217_D3_2007	37	M
10226_D4_2008	36	M
10234_D3_2008	44	M
10287_D4_2008	31	F
10329_D4_2008	23	F
10824_A1_2008	50	F
10833_A1_2008	38	M
11072_E_2008	40	F
11118_D3_2008	68	F
11162_D3_2008	57	M
11176_E_2008	40	M
11177_A1_2008	61	M
11178_A1_2008	48	M
11323_E_2008	41	F
11343_A1_2008	20	M
11436_A1_2008	38	M
11444_E_2008	46	M
11557_E_2008	20	F
11856_A1_2008	42	M

SEQ ID	Age	Gender
11891_E_2008	44	M
11898_A2_2008	27	M
11900_E_2008	36	M
11997_A2_2008	42	M
12125_D3_2008	37	M
12480_A1_2008	63	M
12481_D4_2008	44	M
12997_D3_2008	65	M
13170_A2_2008	35	M
13250_D4_2008	52	M
13426_A1_2008	44	M
13620_A1_2008	36	M
13817_E_2008	35	M
14036_A1_2009	43	F
14041_E_2009	50	M
14280_E_2009	48	M
14410_A1_2009	54	M
14413_A1_2009	32	M
14455_A1_2009	50	M
15071_E_2009	23	M
15237_A1_2009	64	M
15275_A2_2009	43	M
15540_D4_2009	51	M
15687_D4_2009	32	M
15860_A2_2009	42	F
16277_A1_2009	47	M
16660_A1_2009	43	M
17202_E_2009	43	M
17426_E_2009	27	F
17527_D4_2009	59	M
17893_A1_2009	36	F
19607_A2_2009	43	M
20220_E_2009	41	M
20571_E_2010	53	M
20892_A1_2010	40	F
21245_E_2010	41	M
21348_A2_2010	43	M
21639_D4_2010	25	M
22108_A1_2010	58	M
22132_D3_2010	39	M
22965_E_2010	23	F
23297_D3_2010	54	M
23386_E_2010	38	F

SEQ ID	Age	Gender
23412_A1_2011	41	F
25098_E_2011	30	F
25553_E_2011	53	M
28662_A2_2011	55	M
32049_A2_2012	45	M
